# Increases in *Entamoeba histolytica* Antibody–Positive Rates in Human Immunodeficiency Virus–Infected and Noninfected Patients in Japan: A 10-Year Hospital-Based Study of 3,514 Patients

**DOI:** 10.4269/ajtmh.16-0134

**Published:** 2016-09-07

**Authors:** Yasuaki Yanagawa, Naoyoshi Nagata, Koji Watanabe, Kunihisa Tsukada, Katsuji Teruya, Yoshimi Kikuchi, Hiroyuki Gatanaga, Junichi Akiyama, Naomi Uemura, Shinichi Oka

**Affiliations:** ^1^ Department of AIDS Clinical Center, National Center for Global health and Medicine, Tokyo, Japan; ^2^ Center for AIDS Research, Kumamoto University, Kumamoto, Japan; ^3^ Department of Gastroenterology and Hepatology, National Center for Global Health and Medicine, Tokyo, Japan; ^4^ Department of Gastroenterology and Hepatology, National Center for Global Health and Medicine, Kohnodai Hospital, Chiba, Japan

## Abstract

Serological evidence of the epidemiological trends in *Entamoeba histolytica* infection is scarce, especially in nonendemic countries. We aimed to determine the antibody-positive rates over a 10-year period, and compare the trends between human immunodeficiency virus (HIV)–infected and –noninfected patients. We reviewed 3,514 patients who underwent antibody testing during the study periods, which were divided into five annual categories: 2004–2005, 2006–2007, 2008–2009, 2010–2011, and 2012–2013. Anti-*E. histolytica* antibody was assessed by indirect immunofluorescence assay. The antibody-positive rate increased yearly from 2004–2005 to 2012–2013 (*P* < 0.001), although there was no increase in the annual number of antibody tests. This trend was seen among males (18.6–28.3%; *P* < 0.01), females (5.4–28.2%; *P* < 0.01), HIV-infected patients (18.4–26.9%; *P* < 0.001), and non-HIV-infected patients (14.6–36.8%; *P* < 0.001), and HIV-infected men who have sex with men (19.4–29.1%; *P* < 0.001). Among antibody-positive patients, there was a significant increase in the proportion of patients with high (≥ 1,600) titers (0.7–12.9%; *P* < 0.001), whereas this trend was not seen in patients with low (100) or intermediate (200–800) titers (*P* = 0.282 and 0.409, respectively). This large hospital-based study demonstrated that positive anti-*E. histolytica* antibody rates increased over 10 years, even though the annual number of antibody tests remained constant. Moreover, this trend was identified in non-high-risk patients (females and non-HIV-infected patients) as well as in high-risk patients. The proportion of patients with high antibody titers significantly increased among the antibody-positive patients.

## Introduction

Invasive amebiasis, caused by *Entamoeba histolytica*, is one of the most important parasitic diseases responsible for approximately 40,000–74,000 deaths annually around the world.^1^,^2^ This disease remains endemic in developing countries where sanitation infrastructure and health education are inadequate; in these countries, the seropositive anti-*E. histolytica* antibody rates are high.^3^–^12^ On the other hand, there are a limited number of studies on the trends of invasive amebiasis in nonendemic countries.^13^ In Japan, invasive amebiasis is an emerging sexually transmitted parasitic disease, increasing alongside the incidence rates of other sexually transmitted infections (STIs) including human immunodeficiency virus (HIV), chlamydial, and gonococcal infections.^14^,^15^ Previous studies have indicated that HIV infection is a risk factor for invasive amebiasis.^11^,^13^,^16^,^17^ However, epidemiological data on the trends of invasive amebiasis in patients with and without HIV infection are scarce.

Serum anti-*E. histolytica* antibody testing is widely used as an index marker for amebiasis because it is commercially available, inexpensive, noninvasive, and easy to perform.^18^,^19^ When invasive amebiasis is suspected in clinical practice, serological tests for *E. histolytica* antibody are usually the first step due to their noninvasive nature; colonoscopic biopsies of intestinal amebiasis and percutaneous needle aspirations of liver abscess are too invasive to perform in all suspected invasive amebiasis cases. Indirect immunofluorescence (IF) assay is a commercially available method in Japan with excellent diagnostic accuracy.^20^ However, there are no reports on the serological trends of *E. histolytica* based on IF assays. It is essential to prevent the spread of invasive amebiasis if the antibody-positive rates are increasing, especially in nonendemic countries.

The goal of the present study was to determine the antibody-positive rates in clinical practice, and compare the trends between patients with and without HIV infection.

## Materials and Methods

### Study design, setting, and participants.

This is a hospital-based, cross-sectional study. We reviewed 3,514 consecutive adult patients (≥ 18 years old) whose records were from an electronic medical database (MegaOak, NEC, Tokyo, Japan), who had undergone serum anti-*E. histolytica* antibody testing between 2004 and 2013 at the National Center for Global Health and Medicine. The hospital has 900 beds and is the largest referral center for HIV/acquired immunodeficiency syndrome in metropolitan Tokyo. Indications for the antibody testing were as follows: 1) clinical and/or endoscopic findings suspicious for invasive intestinal amebiasis, 2) clinical and/or radiological findings suspicious for amebic liver abscess, and 3) clinically identified STIs. All patients were tested for HIV infection in accordance with hospital policy. We collected data on men who have sex with men (MSM) for HIV-infected patients. This study was approved by the ethics committee of the National Center for Global Health and Medicine Center (approval no. 1424) and was implemented in accordance with the provisions of the Declaration of Helsinki. Patient information was anonymized and deidentified before analysis, and the need for patient consent was waived. This study was institutional review board–approved and patient consent was waived because of retrospective nature.

### Anti-*E. histolytica* antibody test.

The presence of anti-*E. histolytica* antibody was assessed by an indirect IF assay (Amoeba-Spot IF; bioMerieux, Marcy l'Etoile, France), as described previously, which is a common commercial test for the diagnosis of invasive amebiasis in Japan.^21^ Serum antibody titers < 100 were considered negative, whereas titers of 100, 200, 400, 800, 1,600, 3,200, 6,400, and 12,800 were considered positive. Using an antibody titer cutoff of 100 for positive serological tests yielded 89% sensitivity and 87% specificity for the diagnosis of invasive intestinal amebiasis.^20^

### Statistical analysis.

We compared patient characteristics between anti-*E. histolytica* antibody–positive and –negative patients using χ^2^ or Mann–Whitney *U* tests as appropriate. We calculated the antibody-positive rate, and divided the patients into two age groups based on a mean age of 40 years. The study periods were divided into five annual categories: 2004–2005, 2006–2007, 2008–2009, 2010–2011, and 2012–2013. The χ^2^ test for trend was used to determine the trends in proportion by age group, sex, presence of HIV infection, HIV-infected MSM rate, and antibody-positive rate for the five categories. We calculated the ratio of antibody-positive rates in 2004–2005 and compared them to the rates in 2012–2013. We examined whether trends in antibody-positive rates differed by age group, sex, presence of HIV infection, or HIV-infected MSM.

We selected a titer of 100 as the low antibody level because this value was most common in amebic colitis cases.^20^ Other antibody titers (200, 400, 800, 1,600, 3,200, 6,400, and 12,800) were divided in half. Therefore, based on antibody levels, we classified antibody-positive patients into three titer categories: low titer (100), intermediate titer (200–800), and high titer (1,600–12,800). We examined the trends among the three categories during the study period and further analyzed the proportions by age group, sex, and the presence of HIV infection.

A *P* value of < 0.05 was considered statistically significant. All statistical analyses were performed using Stata version 13 software (Stata Corp, College Station, TX).

## Results

The mean age among the 3,514 patients was 39.3 years (interquartile range = 26–53 years), and most were male (93.2%). Of these patients, 3,060 (87.1%) were HIV infected and 454 (12.9%) were non-HIV infected. The mean age of antibody-positive patients was significantly higher than that of negative patients. During the study period, the antibody test has remained constant; 600–780 antibody tests were conducted every 2 years (780 tests in 2004–2005, 777 tests in 2006–2007, 750 tests in 2008–2009, 607 tests in 2010–2011, and 600 tests in 2012–2013). The proportion of MSM significantly decreased from 84% (625/743) in 2004–2005 to 74% (413/561) in 2012–2013.

**Figure 1.**
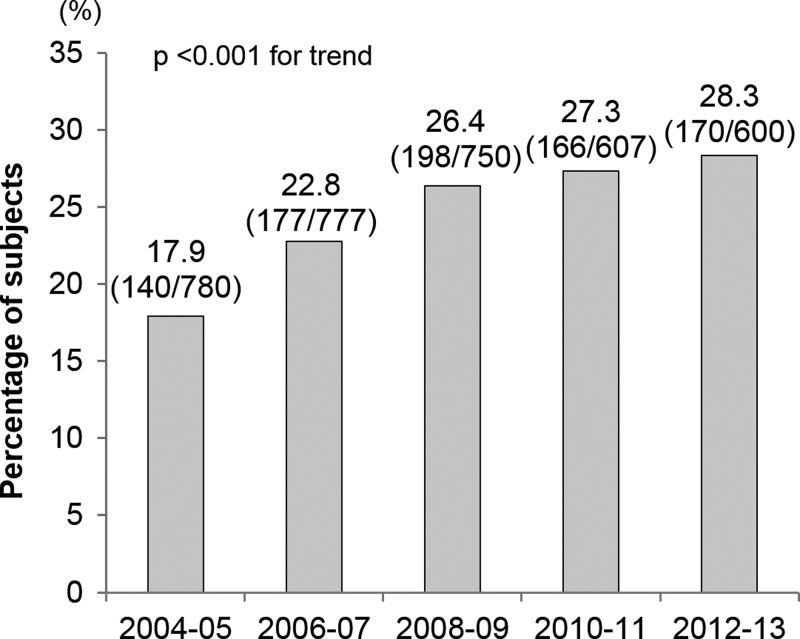
Annual changes in the proportions of anti-*E. histolytica*–positive patients. Note: Values above the bars represent the percentages of positive patients. Values in parentheses above the bars show the number of positive patients/number of patients tested.

The antibody-positive rate increased significantly on an annual basis from 2004–2005 to 2012–2013, and there was an overall 1.58-fold increase from 2004–2005 to 2012–2013 (Figure 1

).

**Figure 2.**
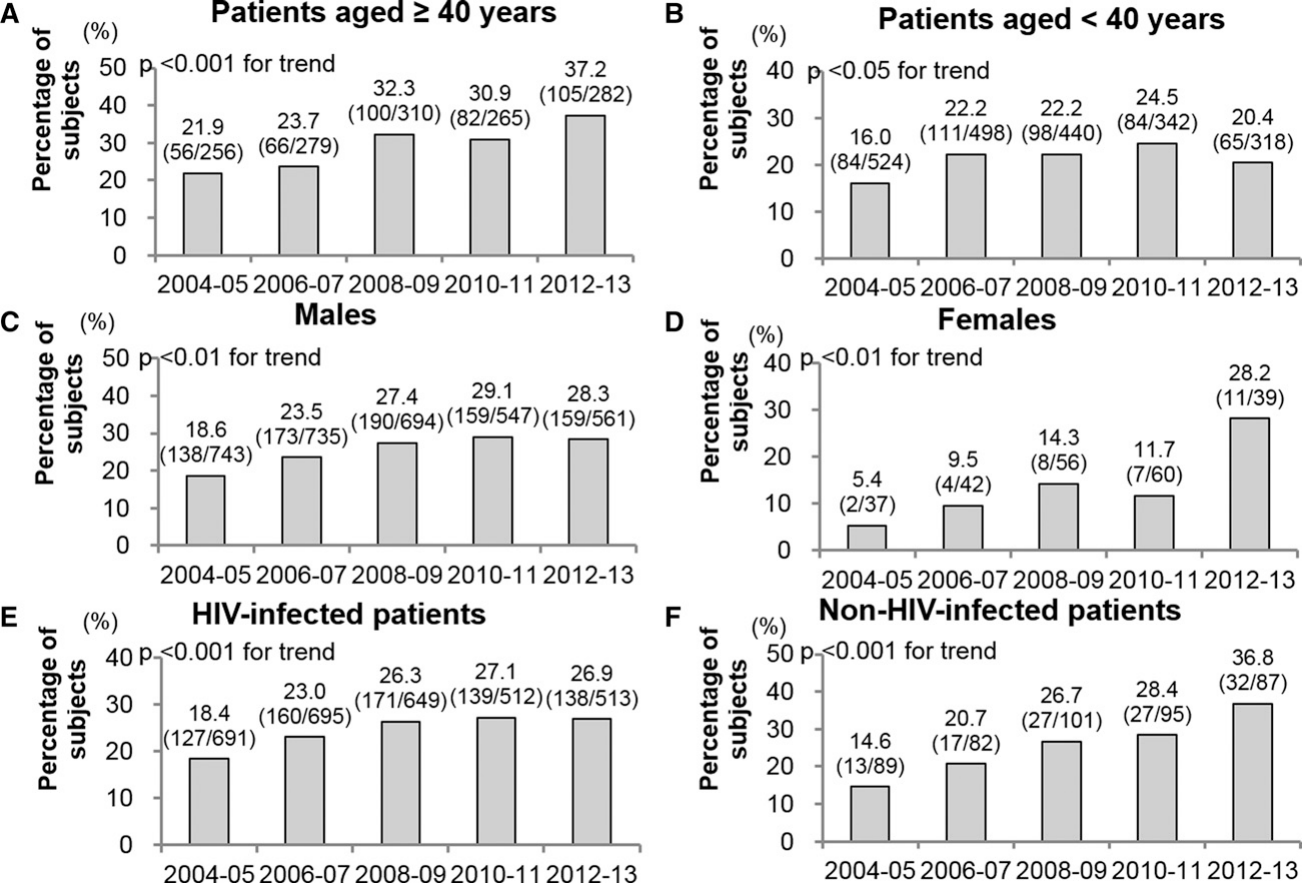
Annual changes in the proportions of anti-*E. histolytica*–positive patients. (**A**) Patients aged ≥ 40 years, (**B**) patients aged < 40 years, (**C**) males, (**D**) females, (**E**) Human immunodeficiency virus (HIV)–infected patients, and (**F**) non-HIV-infected patients. Note: Values above the bars represent the percentages of positive patients. Values in parentheses above the bars show the number of positive patients vs. number of patients tested.

The trends in antibody-positive rates by age groups, sex, and the presence of HIV infection are illustrated in Figure 2

. The proportion of antibody-positive patients aged ≥ 40 years increased significantly each year and 1.70-fold from 2004–2005 to 2012–2013 (Figure 2A). Likewise, the proportion of antibody-positive patients aged < 40 years increased significantly each year and 1.28-fold from 2004–2005 to 2012–2013 (Figure 2B). The proportion of antibody-positive males increased significantly each year and by 1.53-fold from 2004–2005 to 2012–2013 (Figure 2C). Similarly, the number of antibody-positive females increased significantly by year and 5.22-fold from 2004–2005 to 2012–2013 (Figure 2D). Furthermore, the proportion of antibody-positive HIV-infected patients increased significantly by year and 1.46-fold from 2004–2005 to 2012–2013 (Figure 2E); the proportion of positive non-HIV-infected patients showed significant annual increases and grew 2.52-fold from 2004–2005 to 2012–2013 (Figure 2F). The proportion of antibody-positive HIV-infected MSM increased significantly each year: from 19.4% (121/625) in 2004–2005 to 25.4% (153/602) in 2006–2007, 28.1% (153/545) in 2008–2009, 30.4% (128/421) in 2010–2011, and 29.1% (120/413) in 2012–2013. This reflects a 1.50-fold increase from 2004–2005 to 2012–2013.

**Figure 3.**
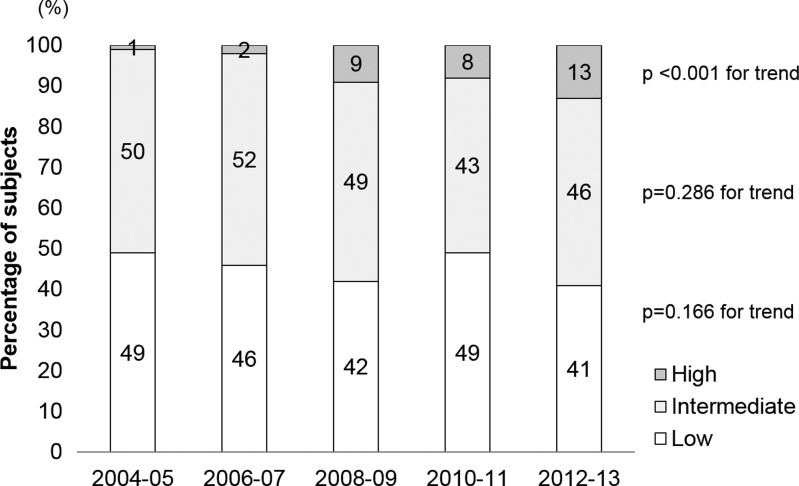
Annual changes in the proportions of three classes based on antibody levels. Note: Antibody level class: low (100 titers), intermediate (200–800 titers), and high (1,600–12,800 titers).

On the basis of antibody levels, antibody-positive patients were categorized as low titers (45.1%), intermediate titers (47.9%), and high titers (6.9%). The trends in the three antibody level categories are shown in Figure 3

. The proportion of high titers increased significantly (*P* < 0.001 for trend) from 0.7% (1/140) in 2004–2005 to 12.9% (122/170) in 2012–2013 with annual increases, and 18.1-fold from 2004–2005 to 2012–2013. There were no significant trends in the low and intermediate titer groups (*P* = 0.282 and 0.409 for trend, respectively).

**Figure 4.**
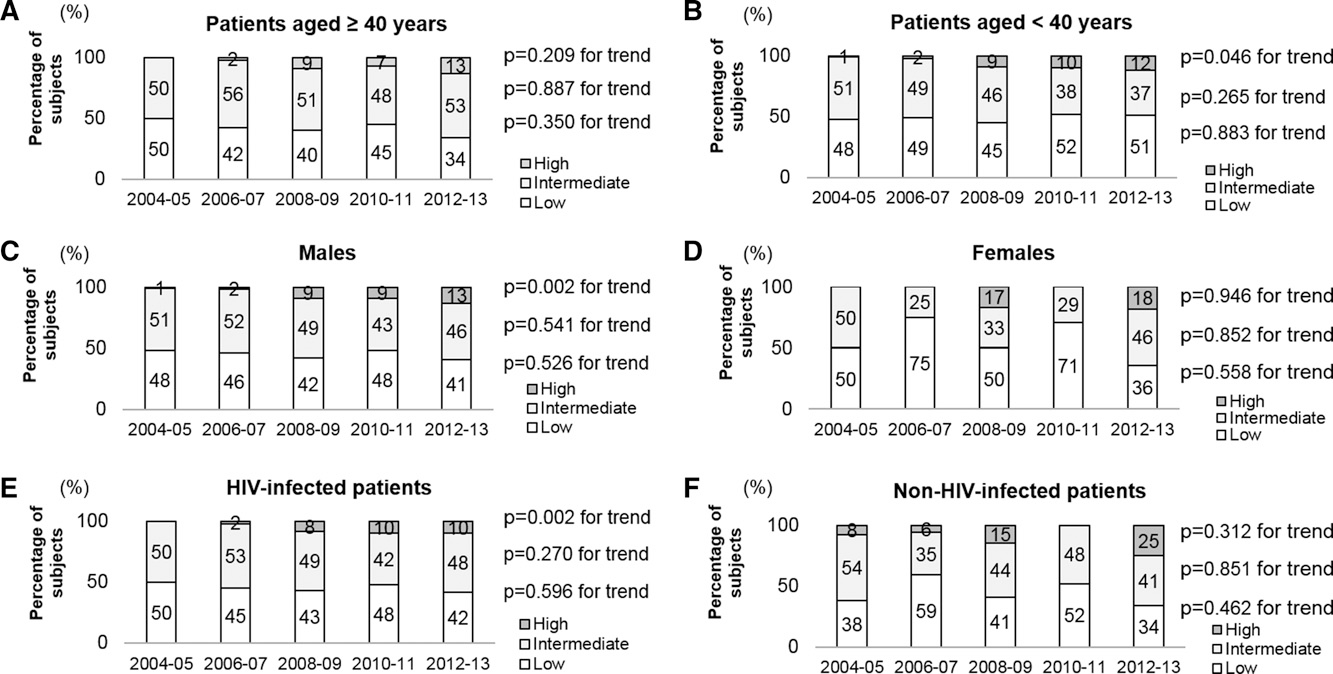
Annual changes in the proportions of three classes based on antibody levels. (**A**) Patients aged ≥ 40 years, (**B**) patients aged < 40 years, (**C**) males, (**D**) females, (**E**) Human immunodeficiency virus (HIV)–infected patients, and (**F**) non-HIV-infected patients. Note: Antibody level class: low (100 titers), intermediate (200–800 titers), and high (1,600–12,800 titers).

The trends in the three antibody level categories by age group, sex, and the presence of HIV infection are shown in Figure 4

. The proportion of patients aged ≥ 40 years with high titers increased significantly from 0% (0/56) in 2004–2005 to 13.3% (14/105) in 2012–2013 (Figure 4A). The proportion of patients aged < 40 years with high titers increased significantly from 1.2% (1/84) in 2004–2005 to 12.3% (8/65) in 2012–2013 (Figure 4B). The proportion of males with high titers also increased significantly from 0.72% (1/138) in 2004–2005 to 12.6% (20/149) in 2012–2013 (Figure 4C). The proportion of females with high titers increased significantly from 0% (0/2) in 2004–2005 to 18.2% (2/11) in 2012–2013 (Figure 4D). The proportion of HIV-infected patients with high titers increased again significantly from 0% (0/127) in 2004–2005 to 10.1% (14/138) in 2012–2013 (Figure 4E). The proportion of non-HIV-infected patients with high titers increased significantly from 7.7% (1/13) in 2004–2005 to 25% (8/32) in 2012–2013 (Figure 4F).

## Discussion

We found that the anti-*E. histolytica* antibody–positive rate increased yearly in over 3,000 patients with a high clinical suspicion, even though the annual number of antibody tests did not increase during the study period. Notably, this trend was found not only among patients who have known risk factors (patients aged < 40 years, male sex, and with HIV infection), but also among those without.^13^,^22^–^26^ Finally, we found that the annual proportion of patients with high (≥ 1,600) titers had increased among all patients and in every subgroup.

We observed an increasing trend in antibody-positive rates in this large-scale hospital-based study using an IF assay. However, since we collected data from the medical records of patients with suspected amebic infection, our results may not accurately reflect the seroprevalence of *E. histolytica* among the general population. Nevertheless, the trend in our study (1.58-fold increase from 2004–2005 to 2012–2013) was consistent with the annual number of reported cases of invasive amebiasis (1.72-fold increase from 2004 [610 cases] to 2013 [1,047 cases]) reported by the national surveillance system in Japan, which requires physicians to notify the Ministry of Health, Labor and Welfare of all invasive amebiasis cases within 1 week. Importantly, the trend in antibody positivity was found not only among high-risk populations such as HIV-infected patients and males, but also among non-high-risk populations such as non-HIV-infected patients and females (Figure 2).

Our group has previously shown that female commercial sex workers (CSW) and those who have sexual contact with CSW represent risk factors for intestinal invasive amebiasis in non-HIV-infected patients.^25^ One report from Japan demonstrated a tendency for higher prevalence of STIs in females with invasive amebiasis.^27^ Moreover, epidemiological studies in Japan have revealed that chlamydial and gonococcal infections are continuously increasing in both sexes.^14^ Considered together, there is a growing concern that sexually transmitted amebiasis is now spreading over to non-MSM and non-HIV-infected populations in our country. It is thought that an effective preventative measure would be difficult to operate because of the increasing number of patients with amebic infections. There are no previous studies indicating an increasing trend in amebic infections in countries where amebiasis is spreading alongside STIs, thus our results highlight the need to comprehend the way in which amebic infections spread among populations, including non-HIV-infected individuals and females.

The spread of sexually transmitted diseases, especially HIV, is expected to rise in Japan, particularly among young adults.^15^ There is an established government scheme for free and anonymous testing and counseling for HIV, syphilis, *Chlamydia*, and gonorrhea at public health centers to improve access. However, there are still no prevention or intervention strategies for sexually transmitted gastrointestinal infections, including invasive amebiasis. We suggest that antiamebic tests be offered to high-risk patients, such as those who are HIV positive. Furthermore, it is important to start early education on sexual health and primary prevention of invasive amebiasis for sexually active teenagers.

Previous studies have shown that antibody levels are usually higher in extraintestinal amebiasis than in invasive intestinal amebiasis.^28^,^29^ Indeed, our group previously reported that antibody levels are higher in patients with extraintestinal amebiasis than in patients with invasive intestinal amebiasis (median titers 400 versus 100; *P* < 0.001).^26^ Although we did not collect data on the organs affected by amebiasis in this study, the proportion of extraintestinal amebiasis cases may have increased in Japan. The present study demonstrated that the proportion of patients with high titers rose significantly among all patients, including those with known risk factors (aged < 40 years, male sex, and HIV infection). This suggests that severe invasive amebiasis, particularly in high-risk patients (e.g., young male HIV-infected patients with amebic liver or intra-abdominal abscesses), will be more common in the future.

Some limitations need to be considered. First, we could not collect sufficient information on sexual behavior or the serological status of other STIs. Second, this study was hospital based, not population based, and antibody testing was performed in clinical practice. Therefore, our results may not be representative of the seroprevalence of invasive amebiasis in the Japanese general population. Despite these limitations, there are several important strengths of this study. First, this study included a relatively large number of patients who underwent antibody testing and long-term observation lasting 10 years. Second, all patients suspected of having invasive amebiasis were tested for HIV infection. Third, indication bias for the antibody testing was less likely to affect our results because the clinical indications for the antibody testing and the institutional background in emergency medicine have remained unchanged for 10 years. Moreover, our seropositive rates showed a similar trend to the annually reported number of cases of invasive amebiasis from national surveillance.

In conclusion, this study showed an increasing proportion of anti-*E. histolytica* antibody–positive patients. This trend was observed in females and non-HIV-infected patients, as well as high-risk patients. Among the antibody-positive patients, the proportion of those with high antibody titers has significantly increased. Amebic infection needs to be recognized as an emerging STI in both HIV-infected and non-HIV-infected patients in Japan. Measures to prevent the spread of amebic infection are urgently needed in Japan.
